# Hyper-inflammation and immunosuppression: redefining sepsis therapy using modern approaches

**DOI:** 10.3389/fphar.2026.1810887

**Published:** 2026-06-17

**Authors:** Mili Prajapati, Vaishnavi Singh, Akash Mishra, Debnarayan Khatua, Minakshi Rana, Anupam Jyoti

**Affiliations:** 1 Department of Life Science, Parul Institute of Applied Science, Faculty of Applied Sciences, Parul University, Vadodara, India; 2 Department of Applied Science and Humanities, Parul Institute of Technology, Parul University, Vadodara, India; 3 Autoimmunity and Inflammation Program, Hospital for Special Surgery at Weill Cornell Medicine, New York, NY, United States

**Keywords:** AI/ML, heterogeneity, hyperinflammation, immunosuppression, precision medicine, sepsis

## Abstract

**Background:**

Sepsis is a life-threatening condition characterized by simultaneous hyperinflammation and immunosuppression, which leads to organ dysfunction and mortality. It is a global health concern, which is estimated to be approximately 166 million cases and 21.4 million global deaths in 2021.

**Aim:**

This review aimed to analyze sepsis epidemiology, pathophysiology, current therapeutic approaches and challenges, and assess the transformational role of modern therapeutic approaches, including precision medicine, and Artificial Intelligence (AI)/Machine Learning (ML) in sepsis.

**Methodology:**

A literature search was carried out across multiple databases, including PubMed, Web of Science, and Scopus. The search was focused on hyperinflammation, immunosuppression in sepsis, current therapy, and future advances.

**Result:**

The review integrated the search data from original articles, review papers, and clinical trials. After careful analysis, we observed that the efficacy of current therapy is limited due to the heterogeneity of sepsis patients, the lack of patient selection based on immune status, timing, and validated biomarkers. Precision methods based on classifying patient genotypes and multi-omics-based biomarkers suggest a potential approach to risk stratification and therapeutic guidance. Concurrently, AI/ML models exhibit improved predictive accuracy and early clinical diagnosis, facilitating early identification and risk stratification.

**Conclusion:**

The integration of AI/ML and precision medicine into sepsis care has the potential to reduce mortality in clinically significant endotypes and allow focused immunomodulatory treatments fulfilling Sustainable Development Goal 3.

## 1 Introduction

The World Health Organization (WHO) views sepsis as one of the top global health concerns as it is one of the leading causes of death in most Intensive Care Units (ICUs), with a high mortality rate (10%–20%) (Rudd et al., 2020). In 2016, the Third International Consensus redefined Sepsis as a “life-threatening organ dysfunction caused by a dysregulated host response to infection” and coined the term Sepsis-3 (Singer et al., 2016). Organ dysfunction can be characterized by an increase in the Sequential Organ Failure Assessment (SOFA) score of two points or more, involving an in-hospital mortality greater than 10% (Singer et al., 2016). The criteria for identifying sepsis in adult patients with suspected infection in emergency department (ED), or general hospital ward settings are determined by bedside clinical score termed quickSOFA (qSOFA) meeting equal or more than any of these two criteria: respiratory rate of 22/min or greater, altered mental status, or systolic blood pressure of 100 mm Hg or less (Ferreira et al., 2001; Singer et al., 2016; Toker et al., 2021). The pathophysiology of sepsis, including dysregulated host response, is complex, dynamic, overlapping, with a highly heterogeneous host-response state. During an infection, innate immune cells recognize the pathogens via pathogen-associated molecular patterns (PAMPs) and pattern recognition receptors (PRRs), which triggers a massive pro-inflammatory cascade. Activation of nuclear factor kappa B (NF-κB) and other signalling pathways led to a “cytokine storm” with high levels of Tumour Necrosis Factor-α (TNF-α), interleukin (IL)-1β, IL-6, and other mediators (Yamamoto et al., 2025). A patient with sepsis requires ICU for a prolonged period; they often develop a chronic illness also called “persistent inflammation, immunosuppression and catabolism syndrome” (PICS), which is characterized by hyperinflammation, immune suppression, dysregulated myelopoiesis, and catabolism (Darden et al., 2021; Wiersinga and van der Poll, 2022). Additionally, the dysregulated host response involves disease tolerance, resilience, resolution and trained immunity (Kox et al., 2026). In parallel, the concept of precision medicine in sepsis is emerging, and researchers are defining clinical subtypes and molecular endotypes so that each patient can receive a therapy matched to their disease profile (Kolodyazhna et al., 2025). ML/AI tools are being developed to detect sepsis earlier and to stratify patients by risk or subtype. Similarly, algorithms that integrate diverse data (clinical records, laboratory biomarkers, even genomics) can identify patient sub-phenotypes in real time (Papareddy et al., 2025). These AI-based approaches may soon support physicians in choosing the right intervention for the right patient at the right time, thus enabling truly personalized care.

In this review, we have discussed epidemiology, pathophysiology, and current therapy in sepsis patients. Furthermore, the role of multi-omics and AI/ML in the precision medicine of sepsis has been explored.

## 2 Epidemiology

Sepsis is a global public health concern due to its high mortality and economic burden (La Via et al., 2024). There were approximately 166 million sepsis cases and 21.4 million global deaths in 2021. There is an increase in sepsis incidence (230%) and mortality (26·3%) in individuals aged 15 years and older since 1990. In addition, the incidence increased from 8.85 million cases in 1990 to 37.1 million cases in 2021 among people aged 70 years and older. Sepsis-related deaths in 2021 from infectious and non-infectious underlying causes increased from 11.8 million to 15.5 million and 4.69 million to 5.81 million since 1990, respectively. Stroke, chronic obstructive pulmonary disease, and cirrhosis were the most prominent non-infectious underlying causes of death, whereas bloodstream infections, including HIV and malaria, as well as lower respiratory infections, including COVID-19, were the leading infectious underlying causes (Gray et al., 2025). The impact of sepsis does not affect geographically; the higher burden rates observed in low- and middle-income countries (LMICs) as compared with high-income countries (HICs) (La Via et al., 2024; Adhikari et al., 2010). An analysis of 170,000 sepsis cases in the United States revealed that 55% of sepsis patients required admission to the ICU (Rhee et al., 2017). Sepsis-induced coagulopathy (SIC) epidemiology is crucial for identifying septic patients who are at risk for overt disseminated intravascular coagulation (DIC) and for predicting mortality rate. A study reported that 332 septic patients were examined for suspected DIC, whereas of the 149 patients diagnosed with overt DIC, 98.7% (147/149) had SIC diagnosis at screening, and out of 49 who developed overt DIC between 2 and 46 were SIC positive, making SIC micro sensitive in detecting those at risk for progression into overt DIC which shows a strong epidemiological correlation of SIC with overt DIC development in septic patients (Iba et al., 2020). These findings imply that SIC could be an important epidemiological marker in septic patients, aiding in the early identification of those at risk for developing overt DIC and potentially helping clinical decision-making by identifying high-risk individuals early in the disease course (Li et al., 2025).

## 3 Pathophysiology: sepsis-induced hyperinflammation and immunosuppression

**FIGURE 1:**
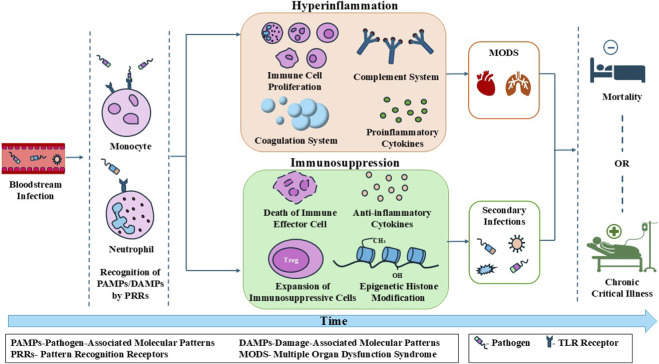
Infection causes a dysregulated host immune response in sepsis. After an infection, the interaction between the complement system and PAMPs/Damage Associated Molecular Patterns (DAMPs) by PRRs activates innate immune cells, which induce the release of cytokines, increase in immune cell proliferation, and their migration to the site of infection and activation of the coagulation cascade. Simultaneously, anti-inflammatory responses, including release of anti-inflammatory cytokines, loss of immune effector cells (T and B cells), expansion of immunosuppressive cells (Treg and Breg cells) as well as epigenetic modifications contribute to immunosuppression and increase the risk of secondary infection.

During sepsis, the immune homeostasis gets imbalanced which is attributed to the hyper-inflammation involving cytokine storm and immunosuppression, which hampers pathogen clearance, tissue repair, and make susceptible to secondary infections. However, the occurrence of both hyper-inflammation and immunosuppression is still debated. Earlier sepsis has been described as a two-phase process, including hyper-inflammation followed by immunosuppression. However, this biphasic distinction is not supported, and both hyper- and hypo-inflammation occur simultaneously (Kox et al., 2026). The complement and coagulation systems, oxygen-free radicals, cytokines, endothelial cells, and leukocytes induce inflammation in sepsis that may cause collateral damage. Uncontrolled activity of pro-inflammatory cytokines such as TNF-α and IL-1β plays a key role in mediating tissue injury (You, 2025). The host’s innate immune system immediately recognizes invading pathogens during the infection. After PAMP recognition by PRRs, neutrophils become activated, produce reactive oxygen species (ROS), and release proteases to amplify inflammation (Takeuchi and Akira, 2010; Nakamori et al., 2021). Additionally, neutrophils form extracellular traps (NETs) composed of histones, DNA, and proteases (Brinkmann, 2018). The severity of sepsis is indicated by the upregulated expression levels of genes contributing to immunosuppression. Patients who survive after the acute hyperinflammatory phase also increased risk of dying from immunosuppression. Acquired immunosuppression in sepsis is caused by metabolic and epigenetic mechanisms resulting in reprogramming of immune cells (Figure 1) (Gao et al., 2025).

### 3.1 Hyperinflammation

#### 3.1.1 Neutrophils

Neutrophils are essential components in the innate immune response and are the first line of defense against microorganisms, and are vital effector cells during inflammation driven by tissue damage (Ramoni et al., 2024). Patients with neutrophil deficiency, such as neutropenia, are more susceptible to microbial and fungal infections because neutrophils have a high capacity and efficacy to detect and eliminate microbial infections (Tak et al., 2017). Sepsis induced the persistent immune dysfunction and inflammation by slowing down the natural programmed cell death (apoptosis) of neutrophils (Zhang et al., 2025). Neutrophils are activated by chemokines that lead to conformational change and initiation of neutrophil integrin molecules such as VLA-4 (CD49D/CD29), MAC-1 (CD11b/CD18), and LFA-1 (CD11Aa/CD18) (Furze and Rankin, 2008). During an infection or tissue damage, inflammatory mediators signal neutrophils to migrate towards the infected area (Bruserud et al., 2023). This migration comprises several stages, beginning with neutrophils migrating along with inflammatory tissues with endothelial tissues, followed by intravascular movement, extravasation, and moving through the interstitial space (Park and Hyun, 2016). Neutrophils generate ROS by activating NADPH oxidase through Raf-MEK-ERK and protein kinase-C (PKC) signalling pathways (Chen et al., 2021). The thrombus formation in sepsis helps in the entrapment of pathogens by NETs and inhibits their propagation (Zhang et al., 2023; Engelmann and Massberg, 2013). Histone networks and DNA fibres in NETs serve as a framework for the attraction of leukocytes, erythrocytes, thrombocytes, and plasma proteins, resulting in positive feedback that promotes and accelerates thrombosis (Li et al., 2020a). Furthermore, extracellular DNA in NETs can promote inflammation and thrombus formation by combining with von Willebrand Factor (vWF) (Sandoval-Pérez et al., 2020; Grässle et al., 2014). NETs enhance the interaction between neutrophils, factor XII (FXII), and initiate the intrinsic coagulation pathway (von Brühl et al., 2012).

#### 3.1.2 Coagulation

The coagulation system consists of endogenous and exogenous pathways, in which the endogenous coagulation system is initiated by FXII, and the exogenous coagulation system is initiated by tissue factor. The soluble fibrinogen is converted into insoluble fibrin to complete coagulation, which is activated by the downstream serine protease-mediated coagulation cascade reactions (He et al., 2024; Levi and van der Poll, 2017). Homeostasis relies on the normal physiological function of the endogenous coagulation system. Suppression of tissue factor-FVII in non-human primates effectively suppresses the coagulation activation, preventing multiple-organ failure (MOF), and reduces the death rate in experimental sepsis produced through intravenous infusion of microorganisms (Welty-Wolf et al., 2001). By cleaving FXI to produce the activated FXI, factor FXIIa initiates the endogenous pathway. In addition, FXIIa initiates the pro-inflammatory kallikrein-kinin system, which results in the contact system in association with the intrinsic coagulation system (Long et al., 2016; Naudin et al., 2017). A significant correlation was observed in sepsis patients with clinical investigations like abnormal coagulation parameters (D-dimer, fibrinogen, and fibrinogen degradation products), prolonged prothrombin time, and activated partial thromboplastin time (He et al., 2024). The excessive coagulation state of blood in the initial stages of sepsis leads to many microvascular thromboses. Severe blood loss develops in the late stage of SIC due to the consumption of fibrinogen and coagulation factors. Most systemic inflammatory responses to infection share a common feature, which is the alteration of coagulation and fibrinolysis in sepsis, and are largely influenced by various cytokines. The thrombin generation is suppressed in endotoxin-exposed chimpanzees and effectively prevents DIC and death in *Escherichia coli*-infused baboons (He et al., 2024; Levi and Poll, 2015; Levi et al., 1994). Except in the cases of severe meningococcemia, the tissue factor is detected on the monocytes in patients suffering from sepsis or in animal models systematically exposed to microorganisms (Levi and Poll, 2015; Østerud and Flaegstad, 1983).

#### 3.1.3 Complement activation

The complement system is the humoral part of the innate immune system, which includes serum proteins, and it is made up of sequential complement protein cleavages that result in a serine protease cascade reaction (Jiang et al., 2024). The complement system plays a critical role in the host response by activation of the classical, alternative, or lectin pathways, which increases the activity of proteases that cleave C3 and C5, resulting in the development of the terminal complement complex (TCC) (De Nooijer et al., 2023; Merle et al., 2015a). TCC causes cellular death by binding to infected cells and cleaving C3 and C5 to produce C3a and C5a, respectively, known as anaphylatoxins, which stimulate the activation of the innate immune response (Merle et al., 2015a; Merle et al., 2015b). Although the complement system is the initial innate immune response to infection, it acts with strong pro-inflammatory actions, which result in tissue damage. Significant pro-inflammatory effects are attributed to the release of allergenic toxins during complement activation, including C3a and C5a (Jiang et al., 2024; Lo et al., 2020). This includes the uptake of leukocytes and increases the vascular permeability, which promotes the body’s defense mechanism (Jiang et al., 2024). In sepsis, the complement system significantly elevates the concentration of factor Bb, C4d, C3a, C5a, and C5b-9 (Wei et al., 2024; Younger et al., 2010). In DIC, the elevated concentrations of C5b-9 in serum were observed, which is associated with severe outcomes, more severe sepsis, and concurrent incidences of DIC (Abe et al., 2020).

#### 3.1.4 Cytokines and chemokines

Cytokines are the signalling proteins that determine the degree and structure of immune responses, and they are the key modulators of inflammation, whereas chemokines act as signalling molecules recognized as chemoattractant cytokines that direct immune cells to the site of infection or injury. The beginning of the acute host response to infections is reported through macrophages and specified by the release of several pro-inflammatory cytokines, which may result in storm (Doganyigit et al., 2022; Takeuchi and Akira, 2010; D'Elia et al., 2013). Inconsistent release of cytokines may result in endothelial dysfunction associated with vasodilation and increased capillary permeability. Moreover, hypotension, hemoconcentration, macromolecular extravasation, and edema in sepsis are connected to the release of inflammatory mediators (Doganyigit et al., 2022). The pro-inflammatory responses are balanced by certain anti-inflammatory cytokines, such as IL-10, transforming growth factor-beta (TGF-β), and IL-4, which maintain immunological equilibrium (Al-Qahtani et al., 2024). Peptide chemokines are present in significant concentrations in the circulation and can minimize local inflammation by desensitization (Doganyigit et al., 2022). TNFα, IL-1, and IL-6 are significant pro-inflammatory cytokines that interact through type I cytokine receptor, whereas G-protein coupled receptors (GPCRs) are used to signal the essential pro-inflammatory chemokine, IL-8 (Turner et al., 2014). A complicated immunological response that changes over time due to pro-inflammatory and anti-inflammatory pathways is initiated by sepsis. Therefore, in septic individuals, immunosuppressive symptoms appear, resulting in uncontrolled inflammatory responses (Doganyigit et al., 2022). The pro- and anti-inflammatory responses in sepsis-induced hyper- and hypo-inflammation are treated and regulated by cytokine-based therapeutic approaches.

### 3.2 Immunosuppression

#### 3.2.1 Apoptosis

Apoptosis is a regulated form of programmed cell death that eliminates the damaged, aged, or unnecessary cells without inducing inflammation (Park et al., 2023; Qian et al., 2024). During the initial phase of immunosuppression, the number of T cells, including both helper T cells and cytotoxic T cells, is low in count due to apoptosis (Sari and Ilyas, 2023; Văsiesiu et al., 2025). Furthermore, dendritic cells become immune-paralyzed and unable to activate T lymphocytes, leading to an imbalance of homeostasis and leading to secondary infection (Zhang et al., 2024). The programmed cell death 1 (PD-1) signalling pathway plays an important role in immune regulation of tumours and autoimmune diseases (Zhong and Yin, 2023). Blocking PD-1 and its ligand PD-L1 has also been explored as a strategy to reverse T cell exhaustion and restore immune function. PD-1/PD-L1 plays a pathological role by altering microbial clearance, the innate inflammatory response, and accelerating apoptosis (Huang et al., 2009). In the acute phase of sepsis, there is a loss of follicular dendritic cells (FDCs) through caspase-mediated apoptosis that occurs in the surviving sepsis mouse model between 36 and 48 h after the onset of sepsis (Tinsley et al., 2003). This appears to be the case in human sepsis as well (Hotchkiss et al., 2002) but does not account for the altered germinal center (GC) function that is present long after the acute exposure, suggesting an additional mechanism is involved. FDCs and GC B cells dysfunction is present in sepsis surviving mice with a likely impairment in the crosstalk that normally regulates GC B cell maturation and FDC function (Rana et al., 2021).

#### 3.2.2 Immuno-metabolism

The progress in immunology led to the establishment of immunometabolism, which focuses on immune cells during homeostasis and infectious inflammatory disease (Kumar, 2019; O’Neill et al., 2016). Organ damage and excessive inflammation are greatly reduced by focusing on the metabolic pathway that involves macrophage immunometabolism at different stages of sepsis. For example, recombinant interferon-γ (rIFN-γ) is used as a potential immunomodulatory agent (Stienstra et al., 2017). An immunometabolism-based cell-specific therapeutic approach proves beneficial in managing sepsis patients depending on their immune status. M1 macrophages that are hyperactivated exhibit different immunometabolism and vary in epigenetic regulatory mechanisms compared to M2 macrophages present at a later stage of sepsis-induced immunosuppression (Kumar, 2018). The nutritional status modulates immune cell function by impacting their metabolic stage during sepsis and infection. The immune cell activation, proliferation, growth, effector response, and restoration of homeostasis is linked to metabolic status and stimulate metabolic reprogramming (Kumar, 2019).

#### 3.2.3 Monocytes and macrophages reprogramming

Monocytes and macrophages are a major component of the host immune system and play a crucial role in managing host immune response during sepsis (Parihar et al., 2010). A study conducted in survivors and non-survivors of sepsis demonstrated that monocyte-derived dendritic cells (MDDCs) underwent caspase-dependent apoptosis, whereas MDDCs from non-survivors followed a necroptotic pathway (Zheng et al., 2024; Raffray et al., 2015). Myeloid-derived suppressor cells (MDSCs) of CD11b^+^Ly6C^high^ cells in the cecal ligation and puncture (CLP) sepsis surviving mouse model showed upregulated pathways enriched in cell cycle, glycolysis, and oxidative phosphorylation, and genes encoding granule proteases. This suggested that MDSCs were metabolically rewired to meet the increased energy demand to support rapid proliferation in sepsis-surviving mice (Watanabe et al., 2024).

#### 3.2.4 Checkpoint regulation

Immune checkpoint molecules are present on lymphocytes and Antigen Presenting Cells (APCs) such as PD-1, PD L-1, CD28, cytotoxic T-lymphocyte antigen-4 (CTLA-4), CD80, CD86, CD40, CD40L, 4-1 BBL, B and T lymphocyte attenuator (BTLA), and T cell-immunoglobulin mucin (Tim) family, play an important role in sepsis by regulating immune response (Busch et al., 2020; Liu et al., 2022). CD28 is the most widely studied immune checkpoint molecule, and its interaction with ligand remains the best characterized pathway, which includes CD28, CTLA-4, and their shared ligand, B7-1(CD80) or B7-2 (CD86, B70) (Bour-Jordan et al., 2011). Inhibition of CD28 protected from septic shock syndrome and death, which is related to upregulation of IL-10 and downregulation of TNF-α production (Wang et al., 1997). Anti-CTLA-4-based immunotherapy has a dose-dependent impact on decreasing sepsis-induced apoptosis in the CLP-induced sepsis animal model. It also influences inflammatory cytokines (Inoue et al., 2011). PD-1 and PD-L1 are important for immunosuppression in sepsis and can be helpful for determining the immunological state and therapeutic target of sepsis (Goyert and Silver, 2010). The TNF family includes both CD40 and CD40L, which mediate the activation and apoptosis of T and B cells and are involved in immune regulation of sepsis (Liu et al., 2022).

#### 3.2.5 Vagus nerve-mediated immunosuppression

It has been apparent now that signals from the brain regulate biological processes in the periphery, including the immune compartment (Chavan et al., 2017). Published reports of the efferent arm of the vagus nerve have shown that vagus nerve signals can suppress monocyte activation through the cholinergic anti-inflammatory pathway (Chavan et al., 2017; Rosas-Ballina and Tracey, 2009; Pavlov and Tracey, 2017). The efferent arm of the vagus nerve releases acetylcholine upon activation. Acetylcholine released by the vagus nerve activates the splenic nerve, triggering the release of norepinephrine in the spleen, which in turn binds to β2 adrenergic receptors expressed on memory CD4^+^ ChAT^+^ T cells that express choline acetyltransferase, the rate-limiting enzyme in acetylcholine. ChAT^+^ T cells, in turn, release acetylcholine, which binds to α7 nicotinic receptors (α7nAChRs) on splenic macrophages to suppress the production of pro-inflammatory mediators. Previously shown that the vagus nerve is constitutively more activated in sepsis-surviving mice than in sham-operated control mice, leading to immunosuppression (Rana et al., 2018). While the cholinergic anti-inflammatory pathway serves a beneficial function in the acute phase of sepsis, its role in the chronic phase of sepsis is more complex, with sustained activation of the vagus nerve contributing to functional impairments, leaving the host at increased risk for subsequent infections (Rana et al., 2018). Recently, it has been shown through electrophysiological recordings that sepsis-surviving mice exhibited higher baseline vagal activity compared to controls and displayed profound dysregulated immune responses (Strohl et al., 2025).

## 4 Current therapy

The present treatment approaches for sepsis include hemodynamic management, host response modulation, and infection control (Vincent, 2022). The mediators and therapeutic targets of hyperinflammation are shown in Table 1, and drugs attenuating immunoparalysis in clinical trials are shown in Table 2.

**TABLE 1:** Pathophysiological factors of hyperinflammation with mediators and therapeutic approaches.

Sr No.	Pathophysiological system	Pathology	Therapeutic approaches	Reference source
1	NETs	NETosis and immunothrombosis: DNA fibres and histone capture RBCs, platelets, and vWF activating factor XII to induce clotting	DNase, PAD4 inhibitors, histone-neutralizing agents to block NETs	Li et al., 2020a; Sandoval-Pérez et al., 2020; von Brühl et al., 2012
2	Coagulation system	SIC and microvascular thrombosis: Activation of TF-FVII (extrinsic) and FXIIa (intrinsic/contact system); modification of fibrinogen to fibrin; metabolism of factors leading to DIC.	Monoclonal antibodies against TF/FVIIa; blockade of FXIIa; suppression of thrombin generation	Levi, 2017; He et al., 2024; Taylor et al., 1991
3	Complement activation	C3/C5 cleavage and TCC: Production of anaphylatoxins (C3a, C5a) and the TCC; leads to opsonization and direct tissue damage	Complement pathway inhibitors: C3 and C5 targeted protease inhibitors	Merle et al., 2015a ; de Nooijer et al., 2023
4	Cytokines and chemokines	Cytokine storm: Uncontrolled release of pro-inflammatory signals (TNF, ILs) and CC/CXC/CX3C/C chemokines; leads to vasodilation, capillary leak, and edema	Cytokine-based treatment approaches; JAK/STAT signalling blockers; anti-inflammatory cytokine regulation (IL-10, TGF-β)	Doganyigit et al., 2022; Schulte et al., 2013

**TABLE 2:** List of drugs attenuating immunoparalysis in clinical trials.

Sr No.	Class	Name of drug	Mechanism of action	Interpretation	Problem associated (side effects)	References
1	Proinflammatory cytokine	IFN-γ	Enhance phagocytosis, increase mHLA DR. Low IFN-γ secretion	No benefit in mortality of ICU randomized clinical trials (RCTs)	Fungal infection, cytokine storm	Delsing et al. (2014)
2	Growth factor	GM-CSF	Reverses sepsis-associated immunosuppression	Improved infection resolution, no mortality benefit	Fever, rash, thrombocytopenia, nausea	Alejandria et al. (2013)
3	Immunostimulatory cytokine	rIL-7	Anti-apoptotic property promotes the proliferation of lymphocytes	Restores lymphocyte level	Fever, injection site reactions	Daix et al. (2023)
4	Natural protein	Immunoglobulin	Neutralizes endotoxins	Supports the immune system	Not reported	Jarczak et al. (2020)
5	Immunomodulating peptide	Tα1	Activates DCs and NK cells, stimulates T-cell proliferation	Reduce mortality, improve mHLA-DR expression.	Not reported	Pei et al. (2018)
6	Stem cells	Mesenchymal stem cells (MSCs)	Regulate immune response, Reduce mortality, Tissue repair	The reversal of immunosuppression has not been clear	No inflammatory storm	Schlosser et al. (2019)
7	Immune checkpoint inhibitor Anti-PD-1 antibody	Nivolumab	Restores immune function	Improves survival, restores cell function Nivolumab: Safe trials	Improves mHDR-DR expression, no safety issue	Hotchkiss et al. (2019)
8	Immunoglobulins	IVIG/IVIGMA (intravenous immunoglobulin), (IVIG enriched with IgM and IgA)	Improve phagocytosis ability, neutralize endotoxin, attenuated neutrophil apoptosis	IVIG does not reduce mortality, IVIGMA potentially reduce mortality	Skin reactions, cardiac effects	Pei et al. (2024)
9	Bacteria/Pathogens	*Mycobacterium* injections (*Mycobacterium indicus pranii*)	Activate Th response, Inactivate antigen-specific immune response	Reduced mortality	​	Kaufmann et al. (2018)

### 4.1 Hemodynamic management

Hemodynamic management supports blood pressure and blood flow in sepsis patients. This usually includes providing fluid resuscitation (IV fluids), injecting vasopressors to constrict blood vessels, which results in high blood pressure, and monitoring blood lactate levels to evaluate tissue oxygenation (Vincent, 2022; Jarczak et al., 2021). The “Hour-1-bundle” is one of the essential elements suggested by the Surviving Sepsis Campaign (SSC), which provides sufficient fluids to maintain the hypovolemic state induced by sepsis, however, the outcomes are not globally effective. There are different Mean Arterial Pressure (MAP) levels based on the patient’s conditions. The SEPSISPAM (Sepsis and Mean Arterial Pressure) study suggests 65–75 mm Hg MAP is sufficient for septic patients, whereas patients with chronic arterial hypertension require 75–85 mm Hg MAP. An optimal level of fluid dose for a pre-existing cardiac patient and septic patients is different, which requires an individual-based treatment approach (Leone et al., 2015). The hypotension or high lactate levels, and fluid resuscitation therapy are the gold standard therapies that have been shown to decrease the mortality rate in septic patients (Jarczak et al., 2021; Rivers et al., 2001; Evans, 2001). Lactate concentration is elevated during aerobic glycolysis in skeletal muscle and not only anaerobic glycolysis even blood pressure, heart rate, and urine output are in normal range (James et al., 1999). Even though it is widely accepted that providing a huge quantity of fluid to septic shock patients is essential for the fast restoration of oxygen and hemodynamics maintenance, sustaining positive fluid balance harms patient outcomes (Acheampong and Vincent, 2015; Marik et al., 2017; van Mourik et al., 2020). Additionally, differing from the current therapy guidelines, advancing research indicates that the early treatment, which initiates with vasopressor medications possibly beneficial for septic patients (Permpikul et al., 2019; Ospina-Tascón et al., 2020).

### 4.2 Modulation of host response

The modulation of the immune response in sepsis is to maintain the balance between pro-inflammatory and anti-inflammatory signals with tissue-protective actions. The broad approaches involve various regulatory targets, like controlling the function of immune cells (neutrophils, macrophages, and lymphocytes), maintaining the coagulation process, and endothelial function to prevent blood vessels from leaking and the formation of microthrombosis, reducing the damage caused by oxidative stress, and rebuilding the overall tissue homeostasis. The overall aim of these interventions is to suppress the threatening hyperinflammation induced by pathogens without exposing the body’s essential immunity and help in enhancing organ recovery (Vincent, 2022). Glucocorticoids are a class of steroids and a common supplement in the treatment of sepsis (hyperinflammation), which target various inflammatory pathways, involving the NF-κB signaling pathway (Heming et al., 2018). Glucocorticoids inhibit the NF-κB activation, which plays a crucial transcriptional factor in inflammation. This results in compromising the host defense mechanism and raises the risk of hemorrhage (Li et al., 2009). In a severe septic shock patient, the corticosteroid is reserved and is often given due to a high requirement for vasopressors like noradrenaline (1 μg/kg/min) to regulate the arterial blood pressure (Venkatesh et al., 2018). Vasopressin is involved in the modulation of host response as the activation of vasopressin earlier in septic shock may improve renal function, support diuresis, reduce fluid requirements, and limit edema formation (Vincent and Post, 2016). Regular corticosteroid administration may results in prevalent metabolic complications including hyperglycemia and hypernatremia (Annane et al., 2017; Chaudhuri et al., 2024). Additionally, HYdrocortisone for PRevention of Septic Shock (HYPRESS) studies failed to prevent the progression from sepsis to septic shock, and large meta-analyses have demonstrated that corticosteroids do not significantly lower long-term mortality (Annane et al., 2017; Liang et al., 2021). Vitamin C is suggested to produce anti-inflammatory effects, specifically on the endothelium. However, massive prospective randomized studies have yielded at best mild benefits, and it is possibly harmful to administer the huge doses of vitamin C to patients with sepsis (Lamontagne et al., 2022). The Lessening Organ Dysfunction with VITamin C (LOVIT) trial reported that high-dose intravenous vitamin C was associated with a higher risk of death or persistent organ dysfunction at 28 days compared to placebo (Lamontagne et al., 2022). Severe clinical complications are associated with complement activation by the C5a–C5aR1 axis. Experimental evidence reveals C5a to be a potent anaphylatoxin and chemotactic attractant of neutrophils and monocytes to sites of infection. This leukocyte (neutrophil) attraction leads to tissue damage, microthrombosis, and inflammation of endothelial cells. Animal experiments in mice indicate attenuation of infiltration in injured organs by organ-specific blockade of the C5a–C5aR1 axis. The blocking is protective in the prevention of excessive pulmonary inflammation and endothelial damage (Vlaar et al., 2022; Afzali et al., 2022; Carvelli et al., 2020). The PANAMO trial showed the safety of the monoclonal anti-C5a antibody Vilobelimab in critically ill COVID-19 patients. The earlier results had already confirmed Vilobelimab to lower serum levels of C5a considerably in these patients (Vlaar et al., 2022). However, no large-scale trial has proven benefit in human sepsis, and there are safety concerns. A major drawback of complement inhibition is the potential for increased susceptibility to secondary infections (McCarthy et al., 2025). Recombinant human IL-1 receptor antagonist (rhIL1RA) is the *in vitro* synthesized analogue of the naturally produced anti-inflammatory cytokine IL1RA. It competitively inhibits by binding to the IL-1 receptor in competition with IL-1α and IL-1β (Meyer et al., 2018; Granowitz et al., 1992; Granowitz et al., 1991). The initial interest in rhIL1RA in the treatment of sepsis emerged from *in vitro* experiments in which IL1β increases vascular permeability and elicits the release of inflammatory cytokines and *in vivo* experiments establishing reversal by IL1RA of febrile vasodilatory shock produced by IL1β (Meyer et al., 2018; Herold et al., 2011; Leff et al., 1994). Biologic blockade of IL-1 (e.g., anakinra, rhIL-1RA) was tested in sepsis, but large trials were negative. A landmark JAMA trial in the 1990s (nearly 1,600 patients) found that there was not a statistically significant increase in survival time for rhIL-1ra treatment compared with placebo. Secondary analyses suggested only a possible dose–response benefit in certain high-risk subgroups (e.g., organ dysfunction, high baseline mortality risk) (Fisher et al., 1994). The Triggering Receptor Expressed on Myeloid Cells 1 (TREM-1) is an immunomodulatory receptor found on innate immune cells, endothelial cells, and platelets, and the plasma levels of sTREM-1 indicate TREM-1 pathway activation (François et al., 2023; Jolly et al., 2021). The 12–amino acid peptide fragment Nangibotide is isolated from TREM-Like Transcript-1 (TLT-1), a TREM-1 family receptor, and is an agonistic TREM-1 ligand binder. Through this action, it regulates amplification of the TREM-1 pathway activation-immune response in acute inflammation (Derive et al., 2012). The ASTONISH Phase 2b trial failed to improve SOFA scores in the overall septic shock population, and the adverse results were similar to placebo, which indicates no clear clinical benefit. Current data suggest that the drug’s efficacy is restricted to a select subgroup (e.g., very high sTREM-1 levels) (François et al., 2023).

### 4.3 Infection control

The key cornerstone of care for sepsis and septic shock is early antibiotic therapy. To ensure maximum effectiveness, awareness of local pathogen epidemiology and the site of potential infection is crucial, as both factors influence prognosis (Strich et al., 2020). Due to large differences in prevalence and resistance patterns of the pathogen between hospitals, regions, and even nations, awareness of the local resistance profile with regular updates is preferred. Treatment empirically should be initiated early with broad-spectrum antibiotics covering the most probable pathogens (Jarczak et al., 2021; Pankey and Sabath, 2004). Excessive use of broad-spectrum antibiotics without proper investigation promotes multidrug-resistant (MDR) and hospital-acquired infections (Martínez et al., 2020). In practice, this is defined rather than based on a hard biological effect. Following an initial loading dose, future dosing must also account for drug characteristics, pharmacokinetics, and pharmacodynamics since organ function is typically impaired in sepsis with fluid resuscitation, transcapillary leakage, hypotension, and renal/liver failure. The treatment is limited to the emergence of antibiotic resistance that requires pathogen identification and targeted therapy (Jarczak et al., 2021).

### 4.4 Immunomodulation therapy targets immunosuppression

Administering immunotherapy for sepsis has become a widely studied topic, and some immunomodulatory drugs are proceeding to clinical trials (van der Poll et al., 2021; Torres et al., 2022; Pei et al., 2024). These include human immune globulin (IVIG–intravenous immunoglobulin), recombinant granulocyte macrophage colony-stimulating factor (rGM-CSF), thymosin alpha 1 (Tα1), recombinant IL-7 (rIL-7), PD-1 or anti-PD-L1 antibody, rIFN-g, and mesenchymal stem cells (MSCs). Its survival benefit remains challenging due to heterogeneity among sepsis patients. IVIG results vary across subgroups and economic regions. It is ineffective in reducing neonatal mortality (RR 0.93, 95% CI: 0.81–1.05, p = 0.24), however, it significantly reduces mortality in adult sepsis patients (RR 0.70, 95% CI: 0.57–0.86, p = 0.0006). Similarly, it was beneficial to high-income (RR 0.89, 95% CI: 0.79–0.99, p = 0.03) and middle-income countries (RR 0.49, 95% CI: 0.28–0.84, p = 0.01) countries but remains ineffective to low-income countries (RR 0.56, 95% CI: 0.27–1.14, p = 0.11) (Pan et al., 2023). GM-CSF is a stimulatory factor that increases the production of neutrophils, monocytes, and macrophages and enhances monocyte survival and immune function. It also enhances phagocytosis and the release of cytokines. Other than rGM-CSF also helps to activate natural immune cells, phagocytic activity, improve bacterial clearance, and reduce unnecessary cell apoptosis (Pei et al., 2024). Many studies show that β-glucan and *Bacillus* Calmette Guerin (BCG) are most frequently used as inducers in the aim to elicit trained immunity (Nankabirwa et al., 2015; Wang et al., 2024). Adult subjects who were given the BCG vaccination showed a 79% decline in infection rates in comparison to subjects given a placebo 1 year after vaccination (Giamarellos-Bourboulis et al., 2020; Wang et al., 2024). However, a 67-year-old patient reported multiple organ failure and septic shock after BCG vaccination, which raises concern about risk factors, diagnosis, and prevention of BCG sepsis (Figueiredo et al., 2013). The state of immunoparalysis among heterogeneous sepsis patients and their response to immunostimulatory drugs can be monitored through biomarkers. The levels of human leukocyte antigen (HLA) DR, present over monocyte and release of TNFα are reduced under *ex vivo* stimulation with LPS, thereby less monocyte responsiveness and are associated with adverse outcomes in sepsis. Hence, both HLA-DR and TNFα levels will be of paramount importance to be used as operational biomarkers to assess the monocyte function in translational critical care research (Joshi et al., 2023; Hall et al., 2011).

Despite the advancements in the therapeutics of sepsis, the current treatments have failed to improve patients' outcomes in clinics in a broader way. Due to the patient heterogeneity, the immune responses vary with time and differ among patients. Additionally, sepsis patients show simultaneous hyperinflammatory and immunosuppressive phases, which make it difficult to administer immunostimulatory and immunosuppressive drugs at the appropriate time to avoid any adverse outcomes (Robey et al., 2024; Slim et al., 2024). The difference in immune response due to infectious pathogens makes it difficult to select appropriate immunomodulators and decide the timing of their interventions (Jain et al., 2024). Furthermore, the lack of validated and established biomarkers classifying the disease severity and indicating the therapeutic responses is another bottleneck which failed to improve desired clinical endpoints (Xiao et al., 2026).

## 5 Precision medicine

### 5.1 Omics technologies

**FIGURE 2:**
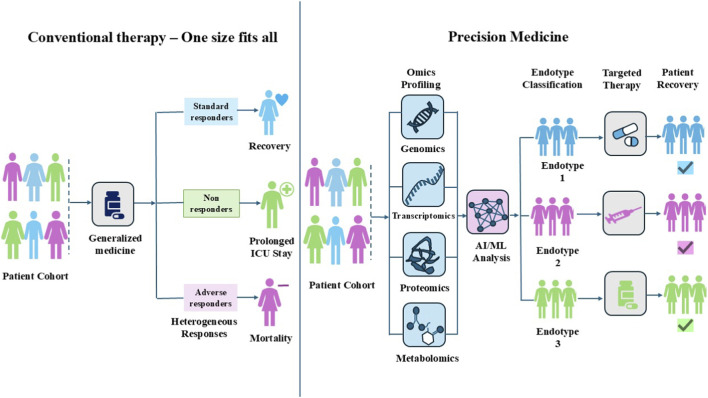
Shows the conventional therapy, where heterogeneous patients are administered with a common treatment, often leads to differential clinical responses, whereas an omics-based approach, followed by AL/ML analysis evaluate the molecular data to determine the specific endotypes and sub-phenotypes, and allows precision medicine to modify the targeted drug therapies to specific subgroups of patients to refine outcomes.

Omics-based modern analytical technologies have been developed over the years to detect distinct biological patterns or other related patterns in response to different therapies in sepsis. Omics-derived biomarkers help to categorize various endotypes and phenotypes in sepsis patients from a clinical point of view (Figure 2). They help to adopt specific therapies to patients according to their unique features (Leligdowicz and Matthay, 2019; Ruiz-Rodriguez et al., 2022). Sepsis clinical presentation and prognosis are associated with genetic variations. An allelic genetic polymorphism that exists in an unalterable state in a population with a frequency that cannot be mutated. This genetic polymorphism has been described in the genes that encode pro-inflammatory and anti-inflammatory cytokines (Nakada et al., 2019; Ruiz-Rodriguez et al., 2022). High occurrence of organ dysfunction in sepsis patients associated with widespread changes in the methylome of their circulating monocytes had aberrant changes in IL-10, and IL-6 (Lorente-Sorolla et al., 2019; Zheng et al., 2020). Epigenetic biomarkers help detect patients with clinical deterioration and unfavorable evolution (Lorente-Sorolla et al., 2019; Zheng et al., 2020; Binnie et al., 2020). Transcriptomics data further classify sepsis patients into various subtypes. The first subtype is characterized by a gene expression profile that significantly increased expression of genes involved in inflammatory and TLR-mediated signalling pathways, associated with a higher prevalence of sepsis (Maslove et al., 2012). Davenport et al. (2016) analyzed the peripheral blood leukocyte global gene expression of 265 critically ill septic patients with community-acquired pneumonia and organ dysfunction (Davenport et al., 2016). The second subtype is associated with adaptive immunity, a lower clinical severity, and a lower mortality rate than the other subtype. Another subtype, which is coagulopathic, was associated with higher mortality and the occurrence of clinical coagulopathy (Wong et al., 2009; Wong et al., 2011). Moreover, metabolomics is used to identify biomarkers, drug activities, or drug-induced toxicity and metabolism. The main objective of metabolomics studies of sepsis patients is to identify prognostic markers, response to therapy, and determine the Systemic Inflammatory Response (SIRS) to treatment (Ruiz-Rodriguez et al., 2022). Proteomics has been explored to identify biomarkers that determine sepsis diagnosis and prognosis. It helps to determine sepsis proteolysis and increase understanding of sepsis pathophysiology and identify new molecules that predict patients' evolution (Ruiz-Rodriguez et al., 2022).

### 5.2 AI/ML

AI and ML approaches promise early recognition, risk stratification, and phenotyping, in sepsis, with the help of retrospective Electronic Health Record (EHR) datasets. These data demonstrate strong discriminatory predictive power for clinical utility. This assists calibration, alert burden, transportability across sites, prevalence-dependent predictive values, external validation, workflow integration, and improves patient-centered outcomes. However, some limitations exist as individual models are unable to replace traditional methods in addition to the heterogeneity in sepsis (Fleuren et al., 2020).

AI/ML based sepsis methodologies are mainly divided into the following categories (i) supervised learning algorithms for classification/regression (e.g., logistic regression, random forests, gradient boosting), (ii) deep learning algorithms for the time course (e.g., RNN/LSTM/temporal convolution), (iii) natural language processing (NLP) models that use unstructured clinical notes, and (iv) reinforcement learning procedures that are focused on the optimization of sequential treatment decisions. Comparative analysis improves prediction period, the presence of features, the quality of labelling (onset-time), and validation from outside (Reyna et al., 2020; Deng et al., 2021). AI/ML models frameworks for sepsis mention in Table 3.

**TABLE 3:** Summary of representative AI/ML models and frameworks in sepsis.

Model/Study	AI approach	Primary data used	Clinical objective	Key contribution	References
Systematic review	Systematic review/meta-analysis	Multiple cohorts	Sepsis prediction performance synthesis	Shows ML can predict sepsis onset (retrospective), highlights heterogeneity and reporting gaps	Fleuren et al. (2020)
InSight	Supervised ML	Vitals (minimal features)	Early sepsis detection	Predicts sepsis onset; robust to missingness	Desautels et al. (2016)
AISE	Interpretable ML	ICU time-series	Early prediction (hours before recognition)	Predicts sepsis 4–12h prior (retrospective)	Nemati et al. (2018)
TREWScore	Risk score/ML	ICU labs + vitals	Septic shock early warning	Targets septic shock prediction with real-time scoring	Henry et al. (2015)
COMPOSER	Deep learning + conformal framework	EHR time-series	Early prediction with low false alarms	Detects “indeterminate”/OOD cases; reduces spurious alerts	Shashikumar et al. (2021a)
DeepAISE	Interpretable deep learning (RNN survival)	ICU time-series	Early prediction + explainability	Time-varying feature contributions to support clinician trust	Shashikumar et al. (2021b)
SERA	Multimodal AI (structured + NLP)	EHR structured + clinical notes	Prediction + diagnosis	Uses unstructured notes to enhance performance	Goh et al. (2021)
PhysioNet/CinC challenge 2019	Benchmarking initiative	Shared clinical dataset	Early prediction evaluation	Standardized task/utility scoring, open comparison	Reyna et al., 2020
AI clinician	Reinforcement learning	ICU trajectories + treatments	Treatment policy learning	Dynamic recommendations for fluids/vasopressors (retrospective)	Komorowski et al. (2018)
TRIPOD+AI/PROBAST+AI/CONSORT-AI/STARD-AI	Reporting/appraisal frameworks	—	Rigor, bias control, reproducibility	Standardizes reporting and bias assessment for clinical AI studies	Ibrahim et al. (2021), Liu et al. (2020), Sounderajah et al. (2025), Collins et al. (2024) , Moons et al. (2025)

#### 5.2.1 Early warning and prognostic models

Primarily ML trained on vital signs acquired through routine monitoring, can predict transitions to septic episodic infections and be robust enough to allow for omissions in the dataset, as shown by the InSight model (Desautels et al., 2016). For example, Targeted Real-time Early Warning Score (TREWScore) has been designed to identify patients at risk of septic shock as early as possible by strongly discriminating and facilitating real-time monitoring within the standard ICU variables (Henry et al., 2015). Moreover, deep learning models have also substantially improved temporal representation learning. For instance, Real-time monitoring using standard ICU variables, such as “Constant LP,” enabled earlier predictions before the realization using real-time data from the ICU, demonstrating the feasibility of ML time-series techniques in critical care.

#### 5.2.2 Robustness, calibration, and reducing false alarms

Robustness, calibration, and the reduction of false positives are important in hospital alert systems. False positives and distribution drift (changes in distribution across hospitals and overtime) are the most severe challenges for clinical applications. Conformal Multidimensional Prediction of Sepsis Risk (COMPOSER) proposed a “learn to say ‘I do not know’” policy by stating that some inputs were not in the training data or were not reliable due to the missingness, sensor errors, and drift, thereby eliminating false alarms (Shashikumar et al., 2021a). Deep Artificial Intelligence Sepsis Expert (DeepAISE) also introduced transparency features by demonstrating the power of each feature in risk prediction on a time basis, thereby supporting ease of use for physicians and enhancing the credibility of the system (Shashikumar et al., 2021b). Calibration is crucial as risk thresholds are often more close to treatment decisions than simply ordering. Along with alert burden, AUROC should also be considered, as high false alerts might curtail usage and offset potential benefits. Therefore, presenting sensitivity, specificity, positive predictive value, and negative predictive value, as well as calibration metrics in clinically relevant populations, will be more conclusive than reporting AUROC.

#### 5.2.3 Going beyond prediction: optimization of treatment itself and of decision support

Apart from its diagnostic properties, AI is also being utilized to decide the treatment regime to be delivered along with their dosages, which is particularly useful in hemodynamic management (fluids/vasopressors). Learning reinforcement has been utilized to learn data-informed policies for sequential decision-making. The principle behind an AI clinician shows how such retrospective trajectories have been used to infer candidate treatment strategies for the study on the concordance between clinician actions and policy recommendations (Komorowski et al., 2018). However, such approaches require careful validation, consideration of causal factors, and prospective evaluation before deployment.

#### 5.2.4 Translation and reporting standards, along with their associated limitations

Most models are limited by the risks of single-centre prediction, variations in sepsis definitions, differences in model validation, and a lack of studies demonstrating outcomes in prospective settings. It is recommended that future AI/ML clinical prediction models be developed and evaluated with increased applicability and improved rigor, using recent reporting and appraisal frameworks, such as Transparent Reporting of a multivariable prediction model for Individual Prognosis Or Diagnosis + Artificial Intelligence (TRIPOD+AI) for prediction model reporting and Prediction model Risk Of Bias Assessment Tool for Artificial Intelligence (PROBAST+AI) for risk-of-bias/applicability information (Collins et al., 2024; Moons et al., 2025). Standards for Reporting Diagnostic Accuracy studies using Artificial Intelligence (STARD-AI) complements the existing guide on diagnostic studies using AI to address concerns about fairness and bias (Sounderajah et al., 2025). AI interventional trials, the Consolidated Standards of Reporting Trials–Artificial Intelligence (CONSORT-AI) extension targets more accurate reporting and interpretative integrity of trial data (Liu et al., 2020; Ibrahim et al., 2021).

#### 5.2.5 From predictive performance to clinical utility

The sepsis detection using AI and ML models achieves high AUROC scores, however their inability to differentiate between conditions demonstrates limited practical value in healthcare settings. A model may distinguish between high- and low-risk patients reasonably well yet remain clinically misleading because it fails to accurately reflect risk through poor calibration. Recent AI-specific reporting frameworks require clinical prediction studies to demonstrate their ability to distinguish cases, but they should also assess calibration, intended use, generalizability, and risk of bias (Collins et al., 2024; Moons et al., 2025). The diagnostic-AI reporting guidance has identified three essential elements which need assessment during the evaluation of clinical AI systems: transparency, bias assessment, and applicability (Sounderajah et al., 2025). The operational effectiveness of an alerting system for sepsis depends on three factors: its positive predictive value, false-positive rate, and the impact of multiple alerts on clinician workflow and trust (Wong et al., 2021). The external validation study of the Epic Sepsis Model shows this problem because the model failed to accurately identify and assess patients who were hospitalized. The current research indicates that transportability remains the primary obstacle because Sepsis Watch achieved strong performance across different health systems through multi-site external validation, yet such results remain uncommon across all models (Valan et al., 2025). A recent systematic review concluded that sepsis prediction studies still need better validation methods, as incomplete validation and reporting make it hard to assess their actual bedside utility (Wang et al., 2025).

The demonstration of clinical usefulness requires evidence of its effects on patient treatment procedures and patient treatment results, which cannot be established through predictive capabilities. The TREWS early warning system evaluation, conducted as a prospective, multisite study, found that healthcare providers who responded to alerts within specific time frames achieved better patient outcomes, including lower rates of death and organ failure (Adams et al., 2022). Yet present research findings on AI technology for sepsis remain incomplete because multiple studies continue to emphasize backwards-looking analysis rather than studying actual outcomes, proper antibiotic use, safe implementation, and fair treatment across different patient groups (Wang et al., 2025; Kim et al., 2025). Future research should assess the full range of evaluation criteria, including components beyond AUROC.

#### 5.2.6 Host-response diagnostics and clinically actionable biomarkers

Sepsis diagnosis is changing due to its dynamic and heterogeneous nature, which hinders clinicians from generalized treatment, hence it is a major bottleneck in personalized therapy. In order to circumvent this, several AI tools have been developed to supplement the existing support for diagnosis, prognosis, patient triage, clinical decision-making, and personalized therapeutic interventions (de Vries et al., 2026; Cajander et al., 2024). Real-time assessment is the primary goal for clinical validation to reduce turnaround time. An EHR based ML score initiates an alert in minutes in comparison to laboratory-based assays, which may take a few hours to days (Islam et al., 2023). For example, FDA-cleared SeptiCyte™ (4-gene RT-PCR) is intended for early diagnosis and differentiating sepsis from non-infectious SIRS with an AUC-ROC 0.82–0.89 for sepsis (Miller et al., 2018). Similarly, Inflammatix’s InSep™ (29-mRNA profile) responds to ED triage of acute infections and distinguishes between bacterial and viral etiology (Safarika et al., 2021). The Epic Sepsis Model (ESM) is also a widely used AI tool for sepsis diagnosis in the ED of hundreds of hospitals in the United States (US). It achieves an AUC of 0.76–0.83, indicating significant predictive ability (Wong et al., 2021). TriVerity (Inflammatix, United States) is another AI-based tool that utilizes gene expression profiling to give a quick report on the probability of bacterial or viral infections and disease severity within 30 min. The TriVerity algorithm was developed using 3,159 patient samples from multiple studies and received FDA approval after accomplishing the prospective SEPSIS-SHIELD study that involved 1,222 patients. It showed an AUC of 0.91 for viral infections and 0.83 for bacterial infections (Liesenfeld et al., 2025; de Vries et al., 2026). The COMPOSER deep learning model allows early intervention by predicting the beginning of sepsis 4–48 h before clinical diagnosis (Shashikumar et al., 2021a).

The workflow begins in ED and ICU with the collection of whole blood in EDTA for cellular tests or PAXgene for RNA analysis. The specimen is examined using SeptiCyte RAPID, TriVerity, and IntelliSep (Balk et al., 2024; Sarani et al., 2024; Figueiredo-Pereira et al., 2025). AI/ML-based algorithms are applied to the raw data to identify the pattern or cellular correlation with the likelihood of infection and severity (Bignami et al., 2025). The IntelliSep Index or SeptiScore translates inputs into a scoring system that categorizes them into risk bands (e.g., Band 1 for low likelihood and Band 3 or 4 for high likelihood), which allows quick interpretation (Berlinger et al., 2025; O’Neal et al., 2024). For example, SeptiCyte has a 4-band SeptiScore (Balk et al., 2024), TriVerity shows probability scores (low/med/high) (Liesenfeld et al., 2025), and IntelliSep provides a 0.1–10 score, divided into three bands (low/medium/high risk) (O’Neal et al., 2024). Based on the outcomes, high-risk results are treated with early antibiotics, strict monitoring, and ICU admission, whereas low-risk result treated with safe de-escalation of empiric antibiotics (Matuszak et al., 2025). Hence, AI-driven integration into the ED enables precise clinical validation and support stratification by recognizing patients most likely to benefit from specific treatments. However, broad clinical adoption requires prospective trials and validation of current gap fulfilment.

Additionally, immune-based classifications involving clinical, biological, and omics data have been performed using unsupervised ML models. Various immunomodulatory therapies, including statins, vasoactive drugs, activated protein C, recombinant drugs, and thrombomodulin, have shown differential effects on these endotypes/subphenotypes (Bruse et al., 2024; Calfee et al., 2018; Sinha et al., 2023; Shen et al., 2025; Neyton et al., 2024; Kudo et al., 2021). This demonstrates that ML-based models have the potential to assist clinicians in optimizing immunotherapy for patient cohorts.

### 5.3 Sepsis subtyping: sub phenotype, and endotype

#### 5.3.1 Sub phenotype

Sub-phenotype is distinguished by a specific set of features not necessarily linked by a common pathophysiological mechanism, but instead by common clinical features (van der Poll et al., 2021; DeMerle et al., 2021; Kolodyazhna et al., 2025). It can be characterized by using readily available clinical and routine laboratory information. Different sub phenotypes have been identified with data obtained from the ED in Sepsis Endotyping in Emergency Care (SENECA) study: α sub phenotype (less organ dysfunction by 33%, 2% mortality); β sub phenotype (more chronic illness and renal dysfunction by 27%, 5% mortality); γ sub phenotype (more inflammation and higher temperature by 27%, 15% mortality); and δ sub phenotype (more hypotension and higher lactate 32%, 32% mortality) (Seymour et al., 2019). Four clusters identified by Knox et al. (2015) using age and SOFA sub scores: shock with elevated creatinine, minimal multi-organ dysfunction syndrome, hepatic disease, shock with hypoxemia and altered mental status (Knox et al., 2015). A retrospective analysis of ProCESS determined phenotypes based on the organ failure patterns that persisted in time (Aldewereld et al., 2022). H1 and H2 are high-risk phenotypes, demonstrating the highest mortality. H1 was constituted as the eldest subgroup characterized by the highest baseline acute physiology, chronic health evaluation, and SOFA score. H1 was further characterized as multi-organ dysfunction, mainly cardiac and respiratory failure (Aldewereld et al., 2022). H2 is the youngest subgroup and has a unique pattern of organ dysfunction consisting of liver failure and coagulopathy, intra-abdominal infection (Aldewereld et al., 2022).

#### 5.3.2 Endotype

In contrast to sub-phenotypes, endotypes are well established for specific pathophysiological mechanisms underlying the clinical presentation. Sepsis patient classification based on endotypes has undergone exponential growth due to advances in genomics, transcriptomics, proteomics, metabolomics, and the availability of datasets combined with data analysis tools (Gordon et al., 2024; Reddy et al., 2020; Kolodyazhna et al., 2025). Davenport et al. (2016) used a transcriptomics study in adults of peripheral blood leukocytes from sepsis patients with community–acquired pneumonia to determine two distinct sepsis response signatures (SRS): SRS1 demonstrated a relatively immunosuppressed character by endotoxin tolerance, T-cell exhaustion, and downregulation of HLA class II (Davenport et al., 2016). SRS1 is related to disturbance of myelopoiesis, leading to an increase in neutrophils and CD4^+^ T cell suppression, which contributes to immunocompromised characteristics (Kwok et al., 2023). The immunocompetent SRS2 endotype is also found in sepsis-oriented faecal peritonitis (Burnham et al., 2017). Molecular endotypes of sepsis based on blood transcriptomes include MARS 1-4. Mars 1 is associated with the worst endotype compared to the others because it has a higher mortality rate than the others. It is characterized by a significant decrease in gene expression related to innate and adaptive immunity cell functions. MARS 2 endotype is characterized by increased expression of genes involved in pathogen recognition, cell growth, cytokine, and mobility pathway, including NF-κB, IL-6, inducible nitric oxide synthase, and N-formyl-methionyl peptide signalling. MARS 3 is relatively low-risk and exhibits increased expression of T-cells. The MARS 4 endotype is also associated with increased expression of genes involved in PRR and cytokine pathways, specifically interferon signalling, RIG1-like receptor and TREM1 signalling activities (Scicluna et al., 2017). The hyperinflammatory phenotype enhances expression of innate immune response genes, while the hypo-inflammatory phenotype enhances expression of adaptive immune response genes (Neyton et al., 2024).

## 6 Conclusion

Sepsis remains a significant global health problem because of its life-threatening and hyperinflammation as well as immunosuppression, leading to multi-organ dysfunction and an ICU mortality rate of 25%. The clinical and molecular heterogeneity in sepsis patients, lack of patient selection based on immune status, timing, and validated biomarkers, limits the effectiveness of uniform treatment strategies. Precision medicine uses multi-omics to classify patients into individual clinical phenotypes and molecular endotypes. AI/ML, particularly ML and deep learning, shows great promise in sepsis diagnosis and by enabling early and more accurate identification of high-risk patients (Yang et al., 2023; Barkas et al., 2025). Over the decades, several AI models have been developed to demonstrate various levels of accuracy, interpretability, and applicability. Reportedly, AUROC values show sensitivity and specificity. Most studied AL systems are DeepAISE, InSight, and Nemati model (Yang et al., 2020; Li et al., 2020b; Shashikumar et al., 2021b; Desautels et al., 2016; Kim et al., 2025). The integration of AI/ML into sepsis care and antimicrobial management represents an advancement in modern medicine, predictive accuracy with AUROC from 0.68 to 0.99, and prediction windows up to 12 h before clinical recognition (Nemati et al., 2018; Pan et al., 2025). Integrating medical records with electronic devices, biomarkers, rapid genomic diagnostics, and metabolomics data has improved predictive performance (Taneja et al., 2017; Taneja et al., 2021).

## 7 Future perspective

The future of sepsis management is based on personalized therapy administered by a combination of precision medicine and AI/ML. Additionally, AI/ML can improve sepsis care by facilitating early detection, precise risk stratification, and multimodal data structure, and possibly even by supporting therapeutic decisions for such cases. However, translating this into bedside practice involves necessary validation and calibration, as well as monitoring for drift, explainability, and adherence to modern reporting standards.
